# Clinical Pharmacology of Clazosentan, a Selective Endothelin A Receptor Antagonist for the Prevention and Treatment of aSAH-Related Cerebral Vasospasm

**DOI:** 10.3389/fphar.2020.628956

**Published:** 2021-02-04

**Authors:** Pierre-Eric Juif, Jasper Dingemanse, Mike Ufer

**Affiliations:** Department of Clinical Pharmacology, Idorsia Pharmaceuticals Ltd., Allschwil, Switzerland

**Keywords:** clazosentan, pharmacokinetics, pharmacodynamics, endothelin, subarachnoid hemorrhage–SAH

## Abstract

Aneurysmal subarachnoid hemorrhage (aSAH) may lead to cerebral vasospasm and is associated with significant morbidity and mortality. It represents a major unmet medical need due to few treatment options with limited efficacy. The role of endothelin-1 (ET-1) and its receptor ET_A_ in the pathogenesis of aSAH-induced vasospasm suggests antagonism of this receptor as promising asset for pharmacological treatment. Clazosentan is a potent ET_A_ receptor antagonist for intravenous use currently under development for the prevention of aSAH-induced cerebral vasospasm. The pharmacokinetics of clazosentan are characterized by an intermediate clearance, a volume of distribution similar to that of the extracellular fluid volume, dose-proportional exposure, an elimination independent of drug-metabolizing enzymes, and a disposition mainly dependent on the hepatic uptake transporter organic anion transport polypeptide 1B1/1B3. In healthy subjects, clazosentan leads to an increase in ET-1 concentration and prevents the cardiac and renal effects mediated by infusion of ET-1. In patients, it significantly reduced the incidence of moderate or severe vasospasm as well as post-aSAH vasospasm-related morbidity and mortality. Clazosentan is well tolerated up to the expected therapeutic dose of 15 mg/h and, in aSAH patients, lung complications, hypotension, and anemia were adverse events more commonly reported following clazosentan than placebo. In summary, clazosentan has a pharmacokinetic, pharmacodynamic, and safety profile suitable to become a valuable asset in the armamentarium of therapeutic modalities to prevent aSAH-induced cerebral vasospasm.

## Introduction

### Endothelin and Endothelin Receptors

Endothelins are highly potent vasoconstrictors displaying a key role in fluid-electrolyte homeostasis as well as cardiovascular and neuronal function (Davenport et al., 2016). These 21-amino acid peptides are synthesized primarily in the endothelium and three isoforms exist (ET-1, -2, -3) that are each encoded by a separate gene. ET-1 is the strongest endogenous vasoconstrictor across multiple organ systems and has been identified in the pathogenesis of, e.g., pulmonary arterial hypertension (PAH) (Chester and Yacoub, 2014), infectious diseases (Freeman et al., 2014) or cancers (Salani et al., 2000a; Salani et al., 2000b; Salani et al., 2002); ET-2 is expressed in the ovary at the time of ovulation when corpus luteum formation begins and induces contraction of ovarian smooth muscles; and ET-3 mediates either vasoconstriction via binding to the ET_B_ receptor or vasodilation via release of nitric oxide (NO) and prostacyclin (Kawanabe and Nauli, 2011). Each ET isoform leads to modulation of vascular tone via binding to ET_A_ or ET_B_ receptors, two G-protein-coupled cell surface receptors (Davenport et al., 2016). Activated ET_A_ and ET_B_ receptors both lead to an increase in intracellular calcium concentration with ET_A_ mediating arterial vasoconstriction, while ET_B_ can have different effects depending on the site of expression, i.e., vasodilation in the endothelium or vasoconstriction in the smooth muscle (Chester and Yacoub, 2014; Davenport et al., 2016). Other signaling pathways have also been demonstrated including epidermal growth factor receptor transactivation (e.g., regulating non-small cell lung cancer cellular proliferation), oxidative stress induction (e.g., cardiovascular fibrosis), rho-kinase (e.g., vasoconstriction), and cyclic adenosine monophosphate (e.g., vasodilation) pathway (Miao et al., 2002; Chester and Yacoub, 2014).

Over the last decades, a number of selective ET_A_ (e.g., ambrisentan, clazosentan, BQ-123, atrasentan), selective ET_B_ (BQ-788), and dual ET_A_/ET_B_ (bosentan, macitentan, aprocitentan, tezosentan) antagonists have been clinically investigated (Okada and Nishikibe, 2002; Sidharta et al., 2015; Houde et al., 2016). Of these, bosentan, ambrisentan, and macitentan were approved for PAH, while other drugs are still in clinical development for the treatment of resistant hypertension (i.e., aprocitentan), kidney diseases (i.e., atrasentan), or cerebral vasospasm (i.e., clazosentan) (Enevoldsen et al., 2020). The development of BQ-123 is no longer pursued.

**FIGURE 1:**
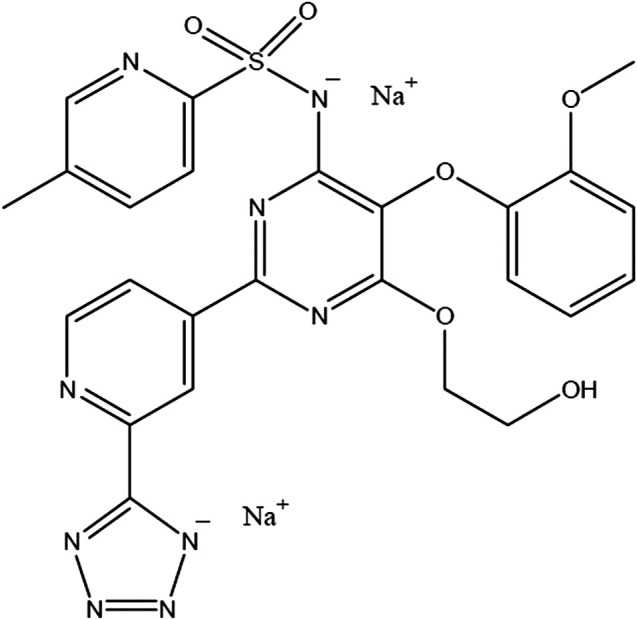
Chemical structure of clazosentan.

This review focuses on the clinical pharmacokinetics (PK), pharmacodynamics (PD), as well as safety and tolerability of clazosentan, which is under development for the prevention of aneurysmal subarachnoid hemorrhage (aSAH)-induced vasospasm (Figure 1) (National Library of Medicine, 2020).

### Pathogenesis of aSAH and Treatment Options of aSAH-Induced Vasospasm

#### Pathogenesis of aSAH

aSAH is mostly caused by the rupture of an aneurysm and represents a significant cause of morbidity and mortality throughout the world (Connolly et al., 2012; Oie et al., 2020), since approximately one quarter of patients with aSAH die, and approximately half of those surviving develop persistent neurological deficits (Nieuwkamp et al., 2009; Connolly et al., 2012).

Cerebral vasospasm leading to reduction in distal blood flow (Zacharia et al., 2010) represents an important factor impacting the global disease burden following aSAH (Lindbohm et al., 2016). Approximately 70% of patients with aSAH develop cerebral vasospasm leading to delayed cerebral ischemia (DCI) or delayed ischemic neurological deficits (DINDs) (Macdonald, 2014; Ayling et al., 2016) and 30% of these patients develop persistent neurological deficits (Rumalla et al., 2018).

Previous studies have identified risk factors contributing to vasospasm-related morbidity and mortality. The extent of subarachnoid hemorrhage at onset as detected by computerized tomography has been shown to correlate with the subsequent development of cerebral vasospasm (Hijdra et al., 1988; Qureshi et al., 2000; Inagawa, 2016; Galea et al., 2017; Jaja et al., 2018; Zanaty et al., 2018). Other risk factors identified were age, history of hypertension, and the SAH severity based on the World Federation of Neurosurgical Societies (WFNS) grading system (Qureshi et al., 2000; Inagawa, 2016; Jaja et al., 2018).

**FIGURE 2:**
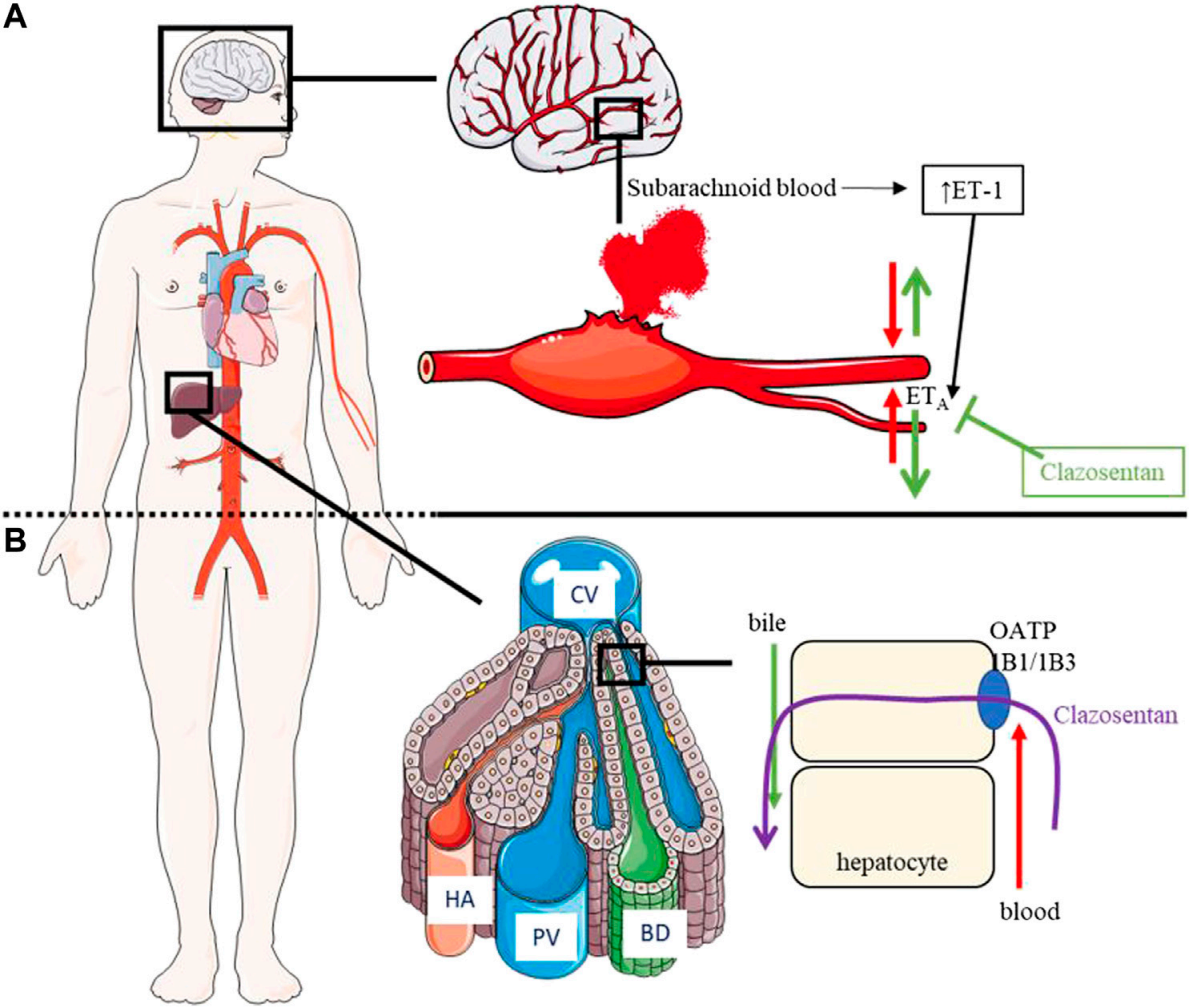
Pathophysiology of aSAH, PD/PK of clazosentan. **(A)**: PD of clazosentan: Following subarachnoid hemorrhage, concentration of ET-1 increases in CSF and ET-1 binds the ET_A_ receptor leading to vasospasm. Clazosentan inhibits the ET_A_ receptor thereby preventing vasospasm. **(B)**: PK of clazosentan: Following intravenous administration, clazosentan enters the systemic circulation and is taken up by OATP1B1/1B3 into hepatocytes followed by excretion into the bile in unchanged form. aSAH, aneurysmal subarachnoid hemorrhage; BD, bile duct; CV, central vein; ET_A_, endothelin subtype A receptor; ET-1, endothelin-1; HA, hepatic artery; OATP, organic anion transporter polypeptide; PV, portal vein.

The etiology of aSAH-mediated vasospasm remains unclear although inflammatory mediators (e.g., cytokines, leukocytes) seem to play a role (Carr et al., 2013). It has also been demonstrated that changes in ET-receptor expression and function in the wall of cerebral arteries may be a causal factor for development of cerebral vasospasm (Behrouz and Sadat-Hosseiny, 2015). In this respect, it has been demonstrated that ET-1 concentrations increase in the cerebrospinal fluid (CSF) of patients with aSAH (Figure 2). Accordingly, messenger ribonucleic acid expression levels of ET_A_ and ET_B_ increased in arteries incubated with hemorrhagic CSF *in vitro* (Thampatty et al., 2011; Cheng et al., 2018). These data suggest that blockade of ET receptors may be beneficial to prevent and treat vasospasm in patients with aSAH.

#### Treatment of aSAH-Induced Vasospasm

A ruptured aneurysm is subject to surgical treatment, namely endovascular coiling (i.e., minimally invasive procedure using a catheter with local release of detachable platinum coils) or microsurgical clipping (i.e., clip fixed around the aneurysm’s neck following craniotomy (Hwang et al., 2012)). Both approaches have advantages and disadvantages, contribute to an improved treatment outcome of aSAH (Connolly et al., 2012), and can be complementary for the prevention of aSAH-induced vasospasm (Taheri et al., 2015).

Both in the United States and in Europe, nimodipine is approved for the prevention of ischemic neurological deficits following aSAH. Several other therapeutic modalities have been employed to prevent or reverse cerebral vasospasm. These include triple H therapy (hypertension, hypervolemia, and hemodilution), balloon and cerebral chemical cerebral angioplasty with intra-arterial injection of vasodilators, or administration of magnesium sulfate, statins, fasudil, ET-1 receptor antagonists, NO progenitors, and sildenafil (Siasios et al., 2013).

Nimodipine, a dihydropyridine, voltage-gated L-type calcium channel blocker, is the only approved treatment of aSAH-associated vasospasm and remains the first-line medical treatment for the management of cerebral vasospasm after aSAH. It had originally been developed as an antihypertensive drug and, only later on, was also developed for treatment of aSAH-associated vasospasm due to some selectivity of its vasodilatory effects for the cerebral vasculature coupled with neuroprotective properties (Tomassoni et al., 2008; Castanares-Zapatero and Hantson, 2011). Nimodipine can also be administered as intra-arterial infusion, which has been shown to reverse established angiographic vasospasm and improve clinical condition (Biondi et al., 2004). However, the i.v. or intra-arterial routes of administration are associated with an increased risk of hypotension compared to oral administration and require repeated or continuous infusion due to a time-limited efficacy (Hanggi et al., 2008). Nimodipine has limited efficacy with respect to major clinical outcomes (e.g., mortality, DCI, and persistent neurological deficits) indicating a major unmet medical need for development of safe and effective treatments of aSAH-mediated vasospasm (Connolly et al., 2012).

Intraoperative local delivery of sustained-release pellets of other dihydropyridines, such as nicardipine, into the subarachnoid space has been shown to improve outcome, however, this drug cannot be used in patients who underwent endovascular coiling due to the invasiveness of the technique (Barth et al., 2011). Also, the development of EG-1962, a nimodipine suspension within a biodegradable polymer, showed promising results which need to be further confirmed (Zussman et al., 2017).

Recent advances of imaging technologies (e.g., transcranial Doppler sonography, computed tomography or magnetic resonance imaging) allow for an earlier diagnosis of aSAH and therapeutic intervention leading to an improvement in survival. This may also be due to a more multidisciplinary approach in the acute management of aSAH involving, e.g., neurosurgeons, radiologists, and endovascular specialists.

## Clazosentan, an ET_A_ Receptor Antagonist: Clinical Pharmacology Profile

### General Background Regarding Clazosentan

Clazosentan (formerly called Ro 61-1790, AXV-034343, and VML 588) is a highly selective ET_A_ receptor antagonist with approximately 1000-fold higher binding affinity to the ET_A_ than to the ET_B_ receptor (Roux et al., 1997). It is highly soluble in aqueous solutions making it suitable for intravenous (i.v.) use.

The PD and efficacy of clazosentan have been explored in a broad spectrum of animal models in rats and cats investigating its effect on cerebral ischemia (Dawson et al., 1999; Bhardwaj et al., 2000), brain lesion caused by cold injury (Gorlach et al., 2001), and vasospasm after aSAH (Schubert et al., 2008). Here, while a curative treatment (i.e., infusion after vasospasm) did not affect neurological outcome (Liu et al., 2018), a prophylactic treatment (i.e., infusion prior vasospasm) with clazosentan prevented vasospasm and hypoperfusion induced by massive SAH (Schubert et al., 2008). Therefore, it was hypothesized, based on these preclinical data, that administration of clazosentan in the acute phase of SAH may reverse perfusion deficits and improve patient outcome.

The PK, PD, and safety of clazosentan have been investigated in more than 2,500 healthy subjects and patients. In healthy subjects, clazosentan was infused at doses ranging from 0.5 to 60 mg/h for up to 72 h, while in patients, doses from 1 to 15 mg/h were infused for up to 14 days. A phase 3 study (REACT, NCT03585270) is currently ongoing in aSAH patients.

### Pharmacokinetics of Clazosentan

**FIGURE 3:**
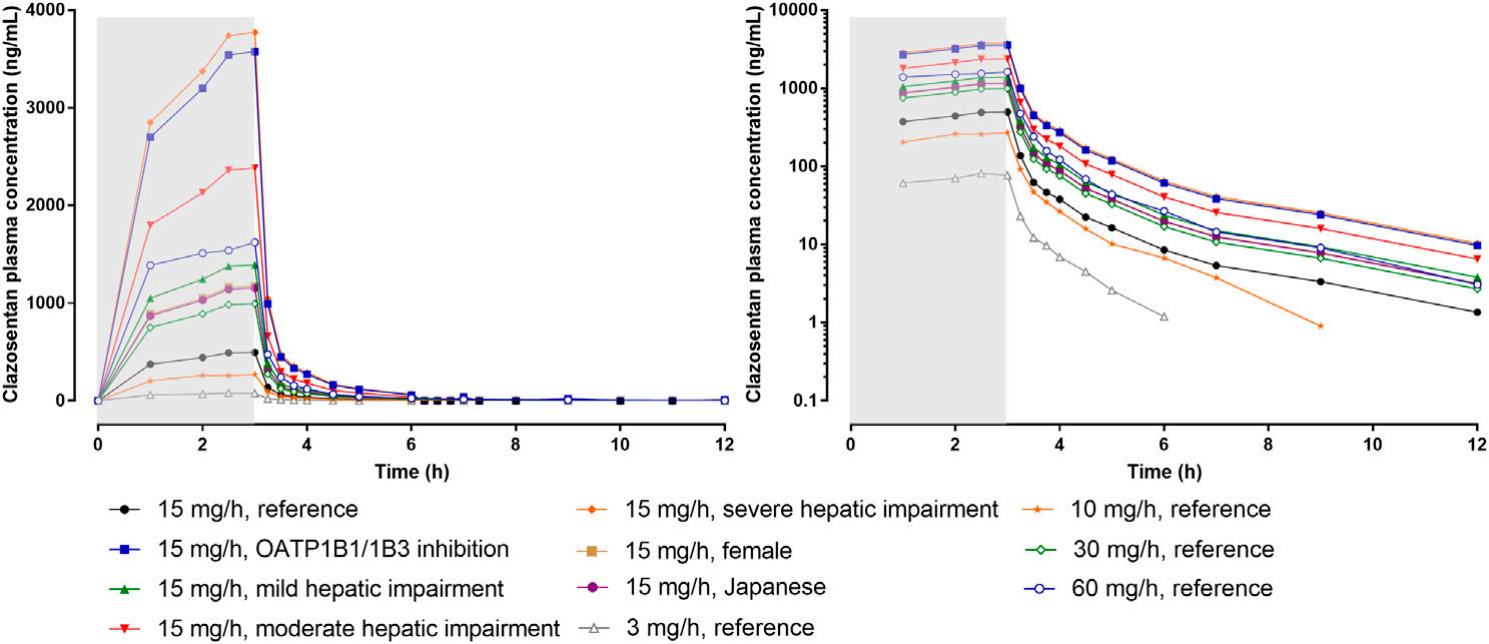
Plasma concentration-time profiles of clazosentan by dose and intrinsic/extrinsic factors in healthy subjects. Data is provided as arithmetic means. The reference subject was a healthy male Caucasian subject. The gray area represents the time of infusion (0–3 h). Left panel: linear scale, right panel: semi-log scale. OATP, organic anion transporter polypeptide.

A schematic representation of the PK and PD of clazosentan is depicted in Figure 2 and the plasma concentration vs. time profile is presented in Figure 3. The PK parameters of clazosentan are provided in Table 1 and Table 2.

**TABLE 1:** PK characteristics of clazosentan.

Maximum tested dose and exposure	Healthy subjects	
	C_max_: 1,637 ± 280 ng/ml (60 mg/h for 6 h)	
	AUC_0-∞_: 9,922 ± 1720 h·ng/ml (60 mg/h for 6 h)	
	AUC_infusion_: 596 ± 85 h·ng/ml (10 mg/h for 3 h)	
	AUC_infusion_: 1849 ± 834 h·ng/ml (30 mg/h for 3 h)	
	AUC_infusion_: 3,692 ± 722 h·ng/ml (60 mg/h for 3 h)	
	aSAH patients	
	C_ss_: 291 ± 104 ng/ml (0.2 mg/kg/h)	
	C_ss_: 667 ± 102 ng/ml (0.4 mg/kg/h)	
Range of linear PK	Dose-proportional PK from 0.5 to 60 mg/h	
Metabolites	Mainly excreted unchanged. No major circulating metabolite. Minor metabolites (<5%) in feces and urine	
Absorption	Absolute bioavailability	100% (by definition)
Distribution	V_ss_	13–32 L
	% Bound	98.4 (97.7–99.5)%
Elimination	Mass balance	Feces: 81%; Urine: 15%
	t_½_	0.7–2.6 h
	CL	32–44 L/h
Intrinsic factors	Age	CL decreases with age (18 vs. 75 years: 48 vs. 31 L/h) Comparable CL between healthy subjects (31–37 L/h) and aSAH patients (28–32 L/h)
	Sex	Similar PK in males and females
	Race	Similar PK in Caucasians and Japanese
	Hepatic impairment	Exposure increases compared to healthy subjects by 1.4-, 2.4-, and 3.8-fold in patients with mild, moderate, and severe hepatic impairment, respectively
	Renal impairment	Similar PK in subjects with severe renal impairment and healthy subjects
Extrinsic factors	Drug interactions	Nimodipine did not affect the PK of clazosentan
		Exposure increased by 3-4-fold upon co-administration of OATP1B1/1B3 inhibitor (rifampin)
	Food effect	Not applicable

aSAH, aneurysmal subarachnoid hemorrhage; AUC_infusion_, area under the plasma concentration-time curve during the infusion; AUC_0-∞_, area under the plasma concentration-time curve from 0 to infinity; CL, clearance; C_max_, maximum plasma concentration; C_ss_, plasma concentration at steady state; i.v., intravenous; OATP, organic anion transporting polypeptides; PK, pharmacokinetic; t_1/2_, terminal elimination half-life; V_ss_, volume of distribution at steady state.

**TABLE 2:** PK parameters of clazosentan by dose in healthy subjects.

Dose and duration of infusion	3 mg/h3 h	10 mg/h3 h	30 mg/h3 h	60 mg/h3 h	60 mg/h3 h	30 mg/h12 h	0.1 mg/h/kg72 h	0.05 mg/h/kg72 h	0.2 mg/h/kg3 h	1, 5, 15 mg/h3 h	1 mg/h3 h	1 mg/h3 h	15 mg/h3 h
Reference	(van Giersbergen and Dingemanse, 2007b)						(van Giersbergen and Dingemanse, 2007a)		(van Giersbergen et al., 2009)	(van Giersbergen et al., 2007)	(Bruderer et al., 2011b)	(Bruderer et al., 2011a)	(Juif et al., 2019)
C_max_	84.6	284	1,029	1,680	1860	798	219	114	422	418	29.8	30.4	519
(ng/ml)	(70.9, 101)	(249, 323)	(889, 1,192)	(1,382, 2043)	(1,541, 2,244)	(675, 944)	(203, 236)	(90.7, 143)	(277, 644)	(356, 491)	(26.1, 33.4)	(24.8, 37.3)	(400, 672)
AUC_0-∞_	205	710	2,535	4,244	9,513	8,092	6,460	4,136	1,300	2038	169	174	1,395
(ng.h/ml)	(179, 235)	(616, 820)	(2,287, 2,811)	(3,448, 5,224)	(7,970, 11,355)	(7,279, 8,996)	(3,701, 11,227)	(2,284, 7,491)	(859, 1967)	(1802, 2,304)	(150, 190)	(144, 211)	(1,106, 1759)
CL	43.9	42.2	35.5	42.4	37.8	41.2	37.7	35.2	30.6	41.2	35.5	34.5	32.3
(L/h)	(38.3, 50.4)	(36.6, 48.7)	(32.0, 39.4)	(34.4, 52.2)	(31.7, 45.1)	(35.9, 47.3)	(32.8, 43.4)	(27.8, 44.5)	(23.9, 39.2)	(36.5, 46.6)	(31.6, 39.8)	(28.4, 41.9)	(25.6, 40.7)
V_ss_	31.4	32.4	27.4	28.4	25.4	23.0	23.7	22.5	13.4	14.3	24.1	19.6	23.5
(L)	(26.4, 37.4)	(27.0, 38.8)	(25.1, 29.8)	(23.5, 34.3)	(20.7, 31.1)	(17.9, 29.6)	(18.2–31.0)	(13.5–37.7)	(10.0, 17.9)	(11.4, 18.0)	(23.0, 25.0)	(15.3, 25.2)	(20.1, 27.5)
t_½_	1.1	1.6	2.5	2.2	2.2	1.9	1.9	1.9	1.4	2.9	2.4	2.0	1.4
(h)	(0.7, 1.9)	(1.2, 2.0)	(1.7, 3.6)	(1.8, 2.6)	(1.5, 3.3)	(1.2, 3.0)	(1.4, 2.5)	(1.1, 3.2)	(1.1, 1.7)	(2.3, 3.8)	(1.9, 3.1)	(1.5, 2.6)	(1.2, 1.6)

AUC_0-∞_, area under the plasma concentration-time curve from 0 to infinity; CL, clearance; C_max_, maximum plasma concentration; t_½_, terminal elimination half-life; V_ss_, volume of distribution at steady-state. Data are presented as geometric mean and 95% confidence interval. Reference Zisowsky et al., 2014: incremental doses were administered consecutively for 3 h each.

#### Absorption and Distribution

In healthy subjects and patients, steady-state plasma concentrations of clazosentan were attained within 3 h after start of a continuous i.v. infusion (van Giersbergen and Dingemanse, 2007b; Zisowsky et al., 2014). In patients, the sparse sampling approach was a limitation for the PK evaluation and required establishing a PK model (Zisowsky et al., 2014).

In healthy subjects, estimates of systemic exposure [maximum plasma concentration (C_max_), concentration at steady-state (C_ss_), and area under the plasma concentration-time curve (AUC)] increased proportionally across the full range of doses (i.e., 0.5–60 mg/h for 6 h) (Table 2). Across doses and studies, clearance (CL) was intermediate (i.e., 31–44 L/h) representing about 35–50% of hepatic blood flow and volume of distribution at steady-state (V_ss_) was similar to that of the extracellular fluid volume (i.e., 13–32 L) indicating limited binding to tissue. The PK of clazosentan are characterized by a low inter-subject variability (van Giersbergen and Dingemanse, 2007b; Zisowsky et al., 2014).

In healthy subjects, clazosentan showed a high degree of plasma protein binding of approximately 98% (van Giersbergen et al., 2009). The blood-to-plasma distribution ratio was approximately 0.65 indicating that clazosentan does not penetrate into human red blood cells (van Giersbergen et al., 2009). Across the different studies in healthy subjects, the plasma concentration–time profile of clazosentan was adequately described by a 2-compartment (van Giersbergen and Dingemanse, 2007b) or 3-compartment model (Bruderer et al., 2011a).

#### Metabolism and Excretion

Experiments in animals have shown that clazosentan was primarily excreted unchanged into feces. In human liver microsomes, a single, minor hydroxylated metabolite was formed by the cytochrome P450 (CYP) isoenzyme CYP2C9 (van Giersbergen et al., 2009).

A mass balance study in humans revealed that about 80% of the administered dose was excreted in feces and 15% in urine (van Giersbergen et al., 2009). Most of the radioactivity measured in plasma and recovered in excreta was unchanged clazosentan indicating that clazosentan is predominantly excreted unchanged in humans. Only few metabolites of low abundance were found in excreta, namely two in feces (about 5 and 1%, respectively, of the administered dose) and four in urine (each <1% of the administered dose).

After the stop of clazosentan infusion, plasma concentrations declined in a biphasic manner with a mean distribution half-life (t_½_) of about 5–7 min and a mean elimination t_½_ ranging from 0.7 to 2.6 h (van Giersbergen and Dingemanse, 2007a; van Giersbergen and Dingemanse, 2007b; van Giersbergen et al., 2007; van Giersbergen et al., 2009; Juif et al., 2019).

#### Intrinsic Factors That May Affect the PK of Clazosentan

Several clinical studies have been conducted to investigate the effect of intrinsic factors on the PK of clazosentan.

Two clinical studies in healthy subjects (van Giersbergen and Dingemanse, 2007a; van Giersbergen et al., 2007) revealed that sex leads to only minor PK differences not considered to be of clinical relevance (i.e., +18% exposure in female subjects). The effect of age on the PK of clazosentan was not investigated in healthy subjects, but in patients with aSAH, the CL of clazosentan decreased with age based on data from large phase 3 studies using a modeling approach (*PK/PD Modeling* section) (Zisowsky et al., 2014).

In an ethnic sensitivity study, similar plasma concentration vs. time profiles and PK parameters were observed in Caucasian and Japanese subjects indicating that the PK of clazosentan are not affected by ethnicity (van Giersbergen et al., 2007).

Two phase 1 studies evaluated the effect of renal (Bruderer et al., 2011b) and hepatic (Bruderer et al., 2011a) impairment on the PK of clazosentan.

In subjects with severe renal impairment, the PK of clazosentan were similar to those in matched healthy subjects as expected based on its minimal excretion in urine and hence dose adjustment is not required in patients with any degree of renal impairment.

In subjects with mild, moderate, and severe hepatic impairment (i.e., Child-Pugh A, B, and C), exposure to clazosentan increased depending on the degree of impairment by approximately 1.4-, 2.4-, and 3.8-fold, respectively, as compared to matched healthy subjects (Table 1). Accordingly, CL decreased and t_½_ increased with increasing degree of impairment, whereas Vss was unaffected. These results were also expected, since clazosentan is mainly excreted unchanged via the bile. Based on these data, no dose adjustment is required in patients with mild hepatic impairment, while in patients with moderate or severe hepatic impairment it is recommended to reduce the dose of clazosentan by 2- and 4-fold, respectively.

A study in aSAH patients revealed that the PK of clazosentan are similar in healthy subjects and patients (van Giersbergen et al., 2008).

#### Extrinsic Factors That May Affect the PK of Clazosentan

Clazosentan was tested for its victim and perpetrator potential with respect to the main human CYP isoenzymes. Regarding its victim potential, the contribution of metabolizing enzymes is not considered relevant, since clazosentan was mainly excreted unchanged in feces and no major metabolites were found. This includes CYP2C9 even though it has been shown to lead to the formation of a single metabolite *in vivo* (*Metabolism and Excretion* section), The perpetrator potential of clazosentan for inhibition of the main human CYP enzymes, i.e., CYP1A2, 2A6, 2B6, 2C9, 2C19, 2D6, 2E1, and 3A4 was evaluated using recombinant CYP isoenzymes expressed in baculovirus-infected Sf9 insect cells with co-expressed CYP reductase. The half-maximal inhibitory concentration (IC_50_) values were >50 µM for all tested enzymes. Considering C_ss_ of 0.64 μM at the expected therapeutic dose and the high degree of plasma protein binding, free clazosentan plasma concentrations are at least 1000-fold lower than the determined IC_50_ values suggesting lack of any relevant perpetrator potential of clazosentan (van Giersbergen et al., 2009).

In terms of drug transporters, clazosentan has been identified *in vitro* as a substrate of organic anion transporter polypeptide (OATP)1B1/1B3 and breast cancer resistant protein (BCRP), but not of other transporters evaluated such as P-glycoprotein (P-gp), bile salt export pump (BSEP), multi-antimicrobial extrusion protein (MATE)-1, MATE-2K, and organic anion transporter (OAT). Its perpetrator potential toward uptake and efflux transporters is considered low in view of IC_50_ values of >20 μM (OATP1B1, OCT1/2, BCRP, P-gp/ABCB1-1, BSEP, MATE-1 and MATE-2K) and >5 μM (OATP1B3, OATP2B1, OAT1, OAT3, and multi-drug resistance protein 2).

As *in vitro* data revealed that clazosentan is a substrate of OATP1B1/1B3 and BCRP, a clinical study in healthy subjects comparing the PK of clazosentan with or without co-administration of the OATP1B1/1B3 inhibitor rifampin was conducted. It revealed 3- to 4-fold greater exposure to clazosentan when co-administered with rifampin as compared to placebo. Accordingly, V_ss_ and CL of clazosentan were decreased by 2.4- and 3.9-fold, respectively, upon co-administration of rifampin, whereas t_½_ was not affected. Rifampin is also known as CYP3A4 inducer when administered as multiple dose (Kalliokoski and Niemi, 2009). Yet, any related confounding of the study results appears unlikely due to the lack of CYP involvement in the metabolism of clazosentan. These study data together with its PK characteristics, i.e., rapid elimination as parent drug in feces, the rate-determining process in the disposition of clazosentan is likely hepatic uptake mediated by OATP1B1/1B3 (Juif et al., 2019).

Although clazosentan is also a BCRP substrate, the impact of BCRP inhibition on its PK was not investigated in humans, since clazosentan has high permeability suggesting a marginal effect of any efflux transporter on its disposition (Poirier et al., 2014). Therefore, hepatic clearance of clazosentan may be mainly driven by hepatic blood flow and independent of BCRP (Rowland and Tozer, 2010).

### Pharmacodynamics of Clazosentan

The principal PD biomarker of ET_A_ receptor antagonism is the concentration of ET-1 in plasma and any derived parameters including ET-1-induced vasoconstriction and modulation of hemodynamics.

#### Effect of Clazosentan on Plasma ET-1 Concentrations

In healthy subjects, ET-1 concentrations in plasma increased during clazosentan infusion. A 2-fold increase in ET-1 concentrations was observed during the 3- and 6-h infusions of 60 mg/h, whereas doses <9 mg/h did not lead to increases in ET-1 concentration. However, there was no clear dose-response relationship observed. The half-maximal effective concentration (EC_50_) was approximately 1,500 ng/ml. An increase in ET-1 concentration was observed at doses of clazosentan >9 mg/h and the onset of this effect was observed right after the start of clazosentan infusion. At 60 mg/h for 6 h and at 30 mg/h for 12 h, steady-state ET-1 concentrations were reached after 3–6 h of infusion. After stop of infusion, plasma ET-1 concentrations fell sharply and matched the time profile of clazosentan plasma concentrations (van Giersbergen and Dingemanse, 2007b). Interestingly, lower doses infused for a longer duration (i.e., doses <9 mg/h infused for 72 h) had no effect on plasma concentrations of endogenous ET-1 indicating that only the plasma concentration of clazosentan at steady-state but not the infusion duration and in turn overall exposure (i.e., AUC) affects ET-1 concentrations (van Giersbergen and Dingemanse, 2007a).

The clazosentan-mediated increase in ET-1 plasma concentrations may appear counterintuitive, as it was suggested that increase in ET-1 concentration is caused by blockade of ET_B_ receptors (Fukuroda et al., 1994) while clazosentan is about 1000-fold more selective for ET_A_ receptors. This suggests that clazosentan may have antagonized not only ET_A_, but also ET_B_ receptors to a certain extent at the highest doses tested.

Though ET-1 concentrations are indicative of the pathogenic alterations of vasospasm and DCI in aSAH patients (Thampatty et al., 2011), ET-1 is not an accepted biomarker of efficacy (Przybycien-Szymanska and Ashley, 2015) as clazosentan-mediated increases in ET-1 concentration may be confounded by the underlying disease.

#### Effect of Clazosentan on Cardiac and Peripheral Hemodynamics

The attenuation of ET-1-induced vasoconstriction by clazosentan was investigated in healthy subjects by administering ET-1 together with clazosentan or placebo. Together with placebo, ET-1 infusion led to alterations of cardiac (cardiac index, stroke index, and pulse wave velocity) and renal (decreased glomerular filtration rate, renal blood flow, renal vascular resistance) function. Clazosentan prevented the cardiac and partially also the renal effects of ET-1 (Vuurmans et al., 2003; Vuurmans et al., 2004).

Clazosentan itself has no effect on cardiac function except for a marginal decrease in blood pressure (BP) (Vuurmans et al., 2003). Antagonism of ET-1 by clazosentan (Vuurmans et al., 2003; Vuurmans et al., 2004) together with clazosentan-mediated increases in ET-1 concentration (van Giersbergen and Dingemanse, 2007b) indicated positive proof-of-mechanism.

Considering that nimodipine is the standard of care for cerebral vasospasm, a drug-drug interaction study in healthy subjects has been conducted (van Giersbergen and Dingemanse, 2008). In this two-way cross over study, nimodipine (60 mg every 4 h for 48 h) was orally administered prior to clazosentan (0.2 mg/kg/h) or placebo infusion. The PK of clazosentan after nimodipine were comparable to historical data supporting the lack of PK interaction between nimodipine and clazosentan.

In the presence of nimodipine, clazosentan elicited no clinically significant BP changes when comparing combined administration of clazosentan and nimodipine to nimodipine alone.

### Safety and Tolerability of Clazosentan

#### Dose Selection

Based on preclinical data, the dose of 3 mg/h was selected as starting dose in the entry-into-human study (van Giersbergen and Dingemanse, 2007b). This dose corresponded to approximately 4% of the pharmacologically active dose (1 mg/kg/h) in the canine model of vasospasm reversal (Roux et al., 1997) and was also more than 100 times below the dose that did not cause any sign of significant toxicity in a 3-days infusion toxicity study in pigs (Idorsia data on file).

In early clinical studies (Vajkoczy et al., 2005; van Giersbergen and Dingemanse, 2007a; van Giersbergen et al., 2009), body-weight adjusted doses were administered (i.e., in mg/kg/h) to achieve similar C_ss_ among patients. Clazosentan does not distribute into fatty tissue (van Giersbergen et al., 2008) as indicated by the V_ss_ of 13–32 L, which corresponds to the extracellular fluid. Therefore, there was no need to correct for body weight and, in later studies, clazosentan was administered on a mg/h basis.

In aSAH patients, doses up to 15 mg/h were administered for up to 14 days. This dose was tolerated and showed efficacy in terms of prevention and treatment of aSAH-induced vasospasm (*Efficacy of Clazosentan* section). In the ongoing phase 3 study, the preventive effect of clazosentan at a dose of 15 mg/h for 14 days is being investigated in patients with aSAH.

#### Adverse Events in Clinical Studies

In clinical studies, headache, nasal congestion, flushing, nasal obstruction, and feeling hot were commonly reported by subjects receiving clazosentan consistent with the mode of action, i.e., vasodilation.

In healthy subjects, clazosentan was infused for a duration of 3–72 h. The maximum total dose administered in 3 h was 180 mg (i.e., 60 mg/h) and associated with headache, nausea, and vomiting as dose-limiting adverse events (AEs) (van Giersbergen and Dingemanse, 2007b). The maximum total dose administered in 72 h was 504 mg (i.e., 0.1 mg/kg/h) that was well tolerated.

In patients with aSAH, the maximum dose was 15 mg/h for up to 14 days that was well tolerated. In phase 3 studies, lung complications, anemia, and hypotension (Macdonald et al., 2011; Macdonald et al., 2012) were more commonly reported after clazosentan than after placebo without any relationship with dose (Song et al., 2019). These lung complications, including pulmonary edema, are commonly observed in aSAH patients and hence may have been confounded by the underlying condition and highlight an important role of proper fluid management in the treatment of these AEs. Hypotension was not observed in healthy subjects, but occasionally in patients that showed a large BP variability likely due to specific triggers in the intensive care unit (ICU) (e.g., stress, surgery), concomitant medications, or underlying disease.

#### QT Liability of Clazosentan

A dedicated thorough QT (TQT) study has been conducted in which clazosentan was infused consecutively for 3 h at 20 mg/h (0–3 h) and at 60 mg/h (3–6 h) resulting in a QT prolongation of regulatory concern (i.e., upper bound of the 90% confidence interval [CI] of ∆∆QTcF exceeded 10 ms). Interestingly, there was a concentration-independent prolongation of the QT interval and presence of QT hysteresis (i.e., delayed effect). Further analysis revealed a relationship between AEs of nausea or vomiting and QT prolongation (Juif et al., 2020). The clinical consequences of this QT liability are probably limited as aSAH patients are under continuous cardiac monitoring in an ICU (Etchegoyen et al., 2017). In addition, clazosentan is intended to be used at the dose of 15 mg/h at which events of nausea and vomiting were rarely observed. Moreover, in previous clinical studies in healthy subjects and patients with aSAH, there were no treatment-emergent AEs of ventricular tachycardia, ventricular fibrillation/flutter, torsade de pointes, and sudden cardiac death (Macdonald et al., 2008; Macdonald et al., 2011; Macdonald et al., 2012).

#### Effect of Clazosentan on BP

In healthy subjects, clazosentan has vasodilatory effects given its mode of action and slightly decreased systolic and diastolic BP. However, this effect was not clinically relevant (i.e., decrease <10 mmHg) nor exposure-related across a broad range of plasma concentrations (90–2000 ng/ml) (van Giersbergen and Dingemanse, 2007b; van Giersbergen et al., 2007; van Giersbergen et al., 2009; Bruderer et al., 2011a; Bruderer et al., 2011b).

#### Effect of Clazosentan on Liver Enzymes

Although increases in liver enzymes and hepatotoxicity have previously been reported for other ET receptor antagonists (Motte et al., 2006), clazosentan did not lead to increases in liver enzyme concentrations even at high doses and with a prolonged duration of infusion in healthy subjects (van Giersbergen and Dingemanse, 2007a; van Giersbergen and Dingemanse, 2007b; van Giersbergen et al., 2009) and patients (Macdonald et al., 2008; Macdonald et al., 2011; Macdonald et al., 2012).

### PK/PD Modeling

A modeling approach has been used to investigate the impact of several covariates on the PK and PD of clazosentan including age, sex, race, weight, body mass index, WFNS score at baseline (i.e., SAH severity), concomitant drug use (e.g., nimodipine), and comorbidities (e.g., hypertension). Source data were gathered from a phase 3 study (CONSCIOUS-2) in patients with aSAH exposed to clazosentan at a dose of 5 mg/h (n = 768) or placebo (n = 389) for up to 14 days (Zisowsky et al., 2014).

CL and volume of distribution were affected by several covariates, however, none of these covariates warrant any dose adjustment.

The population PK analysis also indicated that SAH severity did not affect the PK of clazosentan to a relevant extent and the CL of clazosentan in patients with aSAH (28–32 L/h) was comparable to healthy subjects (31–44 L/h).

Based on the PK/PD model, liver enzyme concentrations were not increased by clazosentan when compared to placebo. This was confirmed in a clinical setting (*Effect of Clazosentan on Liver Enzymes* section).

Based on the PK/PD model, clazosentan led to an exposure-dependent decrease in BP. However, the magnitude was limited as reflected by the slope of the effect of clazosentan on systolic and diastolic BP not significantly different from 0. This suggests no clinically relevant effect on BP, which is in line with data gathered in healthy subjects (van Giersbergen and Dingemanse, 2007b; van Giersbergen et al., 2007; van Giersbergen et al., 2009; Bruderer et al., 2011a; Bruderer et al., 2011b).

In summary, PK and PD data gathered in patients with aSAH were well described by a PK/PD model. There were no covariates affecting the PK of clazosentan to a clinically relevant extent, i.e., none of these covariates warrant any dose adjustment. Safety-related PD variables (i.e., liver enzyme concentrations, BP) were not affected to a clinically relevant extent by clazosentan exposure.

### Efficacy of Clazosentan

The efficacy of clazosentan has been investigated with respect to two main goals in the management of cerebral vasospasm after aSAH, i.e., prevention (start of clazosentan infusion after aSAH) and treatment (start of clazosentan infusion after vasospasm) of vasospasm. In addition, long-term efficacy has been assessed with respect to morbidity and mortality. A total of 3 phase 2 and 2 phase 3 studies have been conducted and approximately 2,300 patients were enrolled.

The first phase 2 study was a double-blind randomized, placebo-controlled study investigating the efficacy of clazosentan in the prevention and treatment of aSAH-induced vasospasm (Vajkoczy et al., 2005). In the prevention part of the study, patients with aSAH received either clazosentan (0.2 mg/kg/h, n = 15) or placebo (n = 17) within 48 h after aSAH for up to 14 days after aneurysm rupture. In an open-label extension treatment part of the study, 19 patients with angiographically confirmed cerebral vasospasm received clazosentan (0.4 mg/kg/h for 12 h followed by 0.2 mg/kg/h for up to 14 days). This study revealed that clazosentan led to a reduced incidence and severity of cerebral vasospasm supporting its preventive effect. In terms of treatment effects, there was some indication of reversal of established cerebral vasospasm by clazosentan (Vajkoczy et al., 2005).

The second double-blind, randomized, placebo-controlled, dose-finding phase 2 study was conducted in Asia. A total of 181 Korean and Japanese patients were enrolled after clipping and received clazosentan (5 mg/h, n = 60; 10 mg/h, n = 60) or placebo (n = 61) for 14 days (Fujimura et al., 2017). Considering the slightly higher exposure in Asian subjects (van Giersbergen et al., 2007) (Figure 3), doses up to 10 mg/h were investigated in Asia and 15 mg/h in other regions. The incidence of vasospasm (i.e., primary endpoint) was significantly lower in patients treated with clazosentan 5 mg/h (38.5%) or 10 mg/h (35.3%) compared to placebo (80.0%).

The third double-blind, randomized, placebo-controlled, dose-finding phase 2 study (CONSCIOUS-1) was conducted to assess efficacy and safety of clazosentan (1 mg/h, n = 107; 5 mg/h, n = 110; or 15 mg/h, n = 96) or placebo (n = 96) for up to 14 days (Macdonald et al., 2008). A total of 409 patients with aSAH were randomized and a dose-dependent reduction in the incidence of moderate or severe vasospasm was observed. There was also a trend for reduced vasospasm-related morbidity and mortality in patients treated with clazosentan. This study helped in selecting the expected therapeutic dose of 15 mg/h (*Dose Selection* section).

The dose of 15 mg/h for up to 14 days was well tolerated in healthy subjects and patients and has been selected as phase 3 dose, i.e., corresponds to the expected therapeutic dose (van Giersbergen and Dingemanse, 2007b; Macdonald et al., 2012; Juif et al., 2019).

In the first randomized, double-blind, placebo-controlled, phase 3 study (CONSCIOUS-2), patients with aSAH secured by surgical clipping were randomized to clazosentan (5 mg/h, n = 768) or placebo (n = 389) for up to 14 days (Macdonald et al., 2011). The dose of 5 mg/h was based on data gathered in the aforementioned phase 2 dose-finding studies. The primary composite endpoint (all-cause mortality, vasospasm-related new cerebral infarcts, DINDs due to vasospasm, and rescue therapy for vasospasm) was met in 21 and 25% of the clazosentan- and placebo-treated subjects, respectively. It was concluded that clazosentan at 5 mg/h had no significant effect on vasospasm-related morbidity and mortality.

In a subsequent randomized, double-blind, placebo-controlled, phase 3 study (CONSCIOUS-3), patients with aSAH secured by endovascular coiling were randomized to clazosentan (5 mg/h, n = 194; 15 mg/h, n = 188) or placebo (n = 189) for up to 14 days (Macdonald et al., 2012). The primary endpoint was identical as in CONSCIOUS-2 and the study was prematurely terminated (577/1,500 of planned patients were enrolled) when data from the CONSCIOUS-2 study became available. The primary endpoint was met in 24, 15, and 27% of the patients treated with, clazosentan (5 mg/h), clazosentan (15 mg/h), and placebo, respectively, indicating that clazosentan significantly reduced post-aSAH vasospasm-related morbidity and mortality at 15 mg/h but not at 5 mg/h.

In both phase 3 studies, the Glasgow Outcome Scale Extended (GOSE) and the modified Rankin Scale both used as markers of long-term clinical outcome did not significantly improve with either dose of clazosentan.

A meta-analysis based on the aforementioned phase 2 and phase3 studies (Vajkoczy et al., 2005; Macdonald et al., 2008; Macdonald et al., 2011; Macdonald et al., 2012; Fujimura et al., 2017) including a total of 1,594 aSAH patients treated with clazosentan and 744 with placebo was conducted (Song et al., 2019). Here, clazosentan in the high-dose group (i.e., >5 mg/h) significantly reduced the incidence of cerebral stroke, vasospasm-related DINDs, and angiographic vasospasm but not in the low-dose group (5 mg/h). While a significant improvement was observed in terms of angiographic vasospasm, there were only marginal differences in rescue therapy and vasospasm-related morbidity and mortality.

An open-label, 2-stage pilot study was performed in patients with aSAH treated with clazosentan (15 mg/h for up to 10 days) after developing moderate-to-severe angiographic vasospasm (Higashida et al., 2019). In contrast to the previous phase 3 studies in which prevention was investigated, the primary efficacy endpoint of this study was the reversal of an existing global cerebral vasospasm in large cerebral artery segments at 3 h after clazosentan initiation. Due to the limited efficacy observed with only three of 11 patients meeting the primary efficacy endpoint, the study was prematurely terminated. However, at 24 h after clazosentan initiation, clear vasodilatory effects were observed in most patients, in particular, in the distal arterial beds of vasospastic cerebral vessels.

Taken together, data gathered in the aforementioned phase 2 and phase 3 studies revealed a preventive effect of clazosentan at doses >5 mg/h in line with preclinical data and a limited treatment effect, i.e., reversal of an existing aSAH-induced vasospasm (Schubert et al., 2008; Liu et al., 2018).

Currently, a phase 3 study (REACT, NCT03585270) is ongoing to evaluate the effects of clazosentan at the expected therapeutic dose of 15 mg/h on the prevention of vasospasm based on worsening of the clinical condition due to DCI as primary endpoint. In this study, the long-term clinical outcomes are also evaluated using the GOSE assessed at 12 weeks after aSAH.

### Clinical Pharmacology, Efficacy, and Safety of Nimodipine

Nimodipine was approved by the Food and Drug administration (FDA) in 1988 and by the European Medicines Agency (EMA) in 1989 for the improvement of neurological outcome by reducing the incidence and severity of ischemic deficits in adult patients with aSAH (Bayer, 2005; Bayer, 2017). It is to be administered orally at a dose of 60 mg every 4 h for 14–21 days after SAH. In Europe, nimodipine is also used as a continuous i. v. infusion, although this is often associated with hypotension (Bayer, 2017). Intra-arterial nimodipine has also shown a promising effect on cerebral vasospasm after aSAH (Bashir et al., 2016).

The PK of nimodipine are characterized by a rapid absorption with a time to reach C_max_ (t_max_) of approximately 1 h, low absolute bioavailability of approximately 13% due to first-pass effect, and a plasma protein binding of >95%. The short t_½_ of 1–2 h requires repeated oral administration of 60 mg nimodipine every 4 h. Except for the low absolute bioavailability (clazosentan has an absolute bioavailability of 100% as administered i.v.), the rapid absorption and elimination characteristics of nimodipine are comparable to those of clazosentan. Also in contrast to clazosentan, nimodipine is highly metabolized, mainly via CYP3A4, and less than 1% is recovered in the urine as unchanged drug (Arbor 2013).

The precise mechanism of action of nimodipine has not been deciphered yet but may involve neuronal as well as vascular effects (Carlson et al., 2020). Nimodipine inhibits calcium ion transfer into smooth muscle cells and thus inhibits contractions of vascular smooth muscle. In animal experiments, nimodipine had a greater effect on cerebral arteries than on arteries elsewhere in the body potentially due to its lipophilicity (Tomassoni et al., 2008; Castanares-Zapatero and Hantson, 2011). Data gathered in large clinical studies in aSAH have shown a favorable effect of nimodipine over placebo on the severity of neurological outcomes (Allen et al., 1983; Pickard et al., 1989; Barker and Ogilvy, 1996; Dorhout Mees et al., 2007) supporting the neuroprotective properties of nimodipine (Tomassoni et al., 2008; Castanares-Zapatero and Hantson, 2011). However, a large number of patients reported nimodipine-induced hypotension that may counteract its benefits (Hajizadeh Barfejani et al., 2019). On the other hand, nimodipine is well tolerated in aSAH patients with AEs mainly affecting the cardiovascular and liver system (Pickard et al., 1989).

An extended-release microparticle formulation of nimodipine that can be administered intraventricularly or intracisternally was developed but failed to show superiority compared to the oral formulation (Hanggi et al., 2017; Hanggi et al., 2019). Further studies are required to develop newer formulations or potential combination of drugs to improve the clinical outcome of aSAH (Carlson et al., 2020).

## Conclusion

ET-1 has strong vasoconstrictive properties via activation of the ET_A_ receptor and as such is involved in the pathogenesis of a broad spectrum of diseases including aSAH-induced vasospasm which represents a major unmet medical need.

Clazosentan, a selective ET_A_ receptor antagonist, is being developed for the prevention of aSAH-induced vasospasm. Its main PK properties are characterized by quick elimination, intermediate clearance, and excretion into the bile mainly in unchanged form, i.e., independent of the activity of drug-metabolizing enzymes. Clazosentan at doses >9 mg/h led to increases in ET-1 concentration. The PD profile of clazosentan is in accordance with its PK profile, i.e., rapid onset and offset of effect.

In patients with aSAH, clazosentan is well tolerated up to the expected therapeutic dose of 15 mg/h, however, lung complications, anemia, and hypotension have been reported. In healthy subjects, the dose-limiting AEs were headache, nausea, and vomiting. Efficacy of clazosentan has been demonstrated at doses >5 mg/h with respect to the prevention of vasospasm based on morbidity and mortality data in several phase 2 and 3 studies. However, there is limited evidence of efficacy in the treatment of existing vasospasm at any dose investigated.

In summary, clazosentan has a PK, PD, and safety profile suitable to become a valuable asset in the armamentarium of therapeutic modalities to prevent cerebral vasospasm after aSAH.
